# Supplementary material for: Effect of Visual Booklets to Improve Bowel Preparation in Colonoscopy: Systematic Review with Meta-Analysis
Source: J Clin Med. 2023 Jun 29;12(13):4377. doi: 10.3390/jcm12134377 (PMC10342745; doi:10.3390/jcm12134377)
Supplement: Supplementary file 1 [file jcm-12-04377-s001.zip › jcm-2429224-supplementary.pdf]

Supplementary Figure S1

Study or Subgroup	A	B	C	D	E	F	G
Calderwood 2011	+		+	−	+	+	+
Ergen 2016	+		+	+	+	+	+
Guardiola-Arevalo 2019	+		+	+	+	+	+
Ozkan 2020	+		−	−	+	−	
Spiegel 2011			+	+	+	+	+
Tae 2012	+		+	+	+	+	+

Risk of Bias (columns A–G)

<u>Risk of bias legend</u>

(**A**) Random sequence generation (selection bias)
(**B**) Allocation concealment (selection bias)
(**C**) Blinding of participants and personnel (performance bias)
(**D**) Blinding of outcome assessment (detection bias)
(**E**) Incomplete outcome data (attrition bias)
(**F**) Selective reporting (reporting bias)
(**G**) Other bias
